# Learning from the radiological findings of dropped gall stone and/or appendicolith (its complication and management strategy)

**DOI:** 10.1259/bjrcr.20180096

**Published:** 2019-03-18

**Authors:** Yasir Jamil, Dr Nicholas Reading

**Affiliations:** 1 Department of Clinical Radiology, Whipps Cross University Hospital, London, UK

## Abstract

This case report the patient presented with intra-abdominal abscess with the past surgical history of laparoscopic cholecystectomy and appendectomy. Being a radiologist, it is important to keep patient’s previous surgical interventions in mind as it can change the management options.

## Clinical presentation

A 53-year-old Caucasian female with a background of laparoscopic cholecystectomy (LC) and appendectomy 18 and 32 years ago respectively presented with 6 months’ history of ongoing right flank pain worsening over the last few weeks. On clinical examination, there was a fluctuant and erythematous swelling in the right flank.

## Differential diagnosis

- Renal colic
- Urinary tract infection
- Pyelonephritis
- Acute abdomen
- Sepsis
- Psoas abscess

## Investigations/Imaging findings

CT of the abdomen and pelvis demonstrated intra-abdominal abscess extending into the right flank/subcutaneous plane. The collection was predominately retroperitoneal and extended superiorly to the level of the midpole of the right kidney and inferiorly into the right iliac fossa.

**Figure 1.**
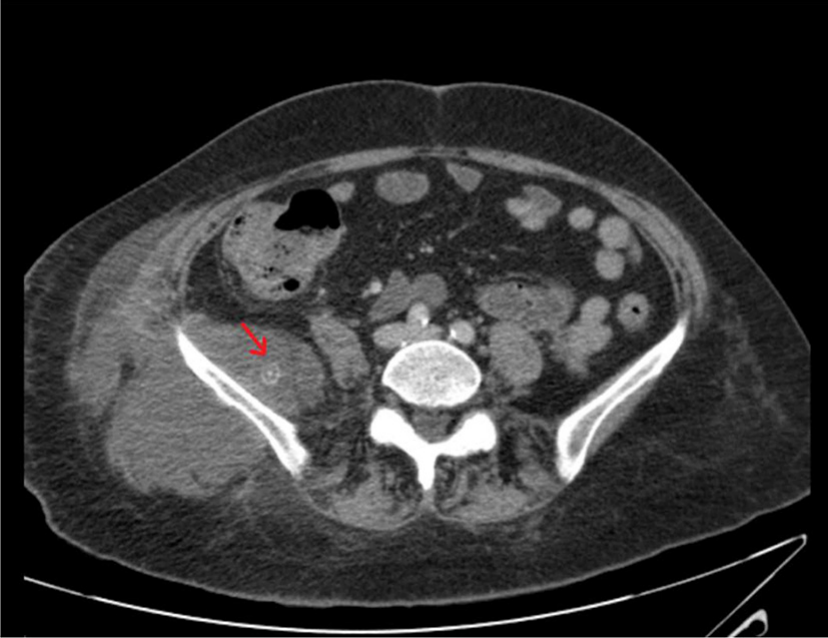
CT abdomen and pelvis (axial view) shows frank abscess with 9 mm well-defined lamellated calculus (red arrow).

**Figure 2.**
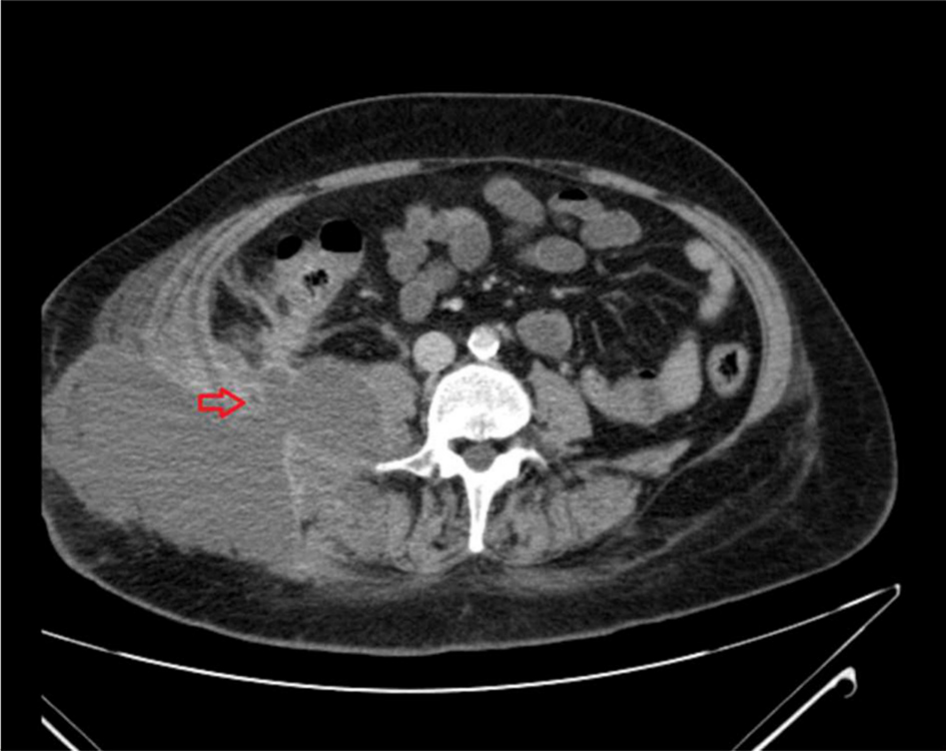
CT abdomen and pelvis (axial view) shows intra-abdominal abscess extending into the right flank/subcutaneous plane (red arrow).

**Figure 3.**
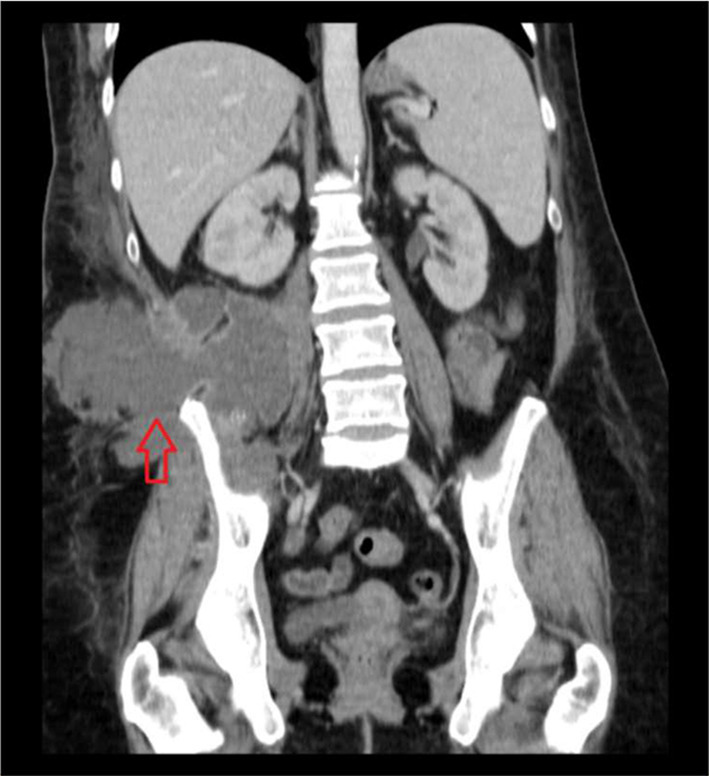
CT abdomen and pelvis (coronal view) shows intra-abdominal abscess extending into the right flank/subcutaneous plane (red arrow).

The Frank abscess also demonstrated a 9 mm well defined lamellated calculus (Figures 1–3).

No prior imaging was available.

### Outcome, follow-up and discussion

Dropped gall stones (DGs) are uncommon complication of LC.^1,2^ It may present with non-specific clinical symptoms such as fever, abdominal pain due to abscess, adhesion, fistula formation..^3,4^ Time to presentation varies from days to months or even years after the procedure.^3^ These abscesses are usually found in the right upper quadrant, however this may not always be the case.^2,3,5^

As far as the pathophysiology of DGs is concerned, over the period it is associated with slow granulomatous response which is profoundly related to pigment stones because of the bilirubin polymer. Clinical implications if the DGs are not extracted can cause other rare complications which are associated with migration of DGs to nearby structures such as urinary tract, presenting as ovarian cholelithiasis, causing chronic pelvis pain. Moreover, DGs can also erode large bowel and cause complications like adhesions, obstructions, pelvis actinomycosis to name but a few.^6,7^

Another less frequent cause of right flank abscess can be appendicolith. In our case study, the patient also has a childhood history of appendectomy. The size of the focus <1 cm within the abscess and identical imaging findings to the DGs also favours the possibility of dropped appendicolith.^3,8^ Dropped appendicolith is rare modality as compared to DGs and often present as intra-abdominal abscess.^9^

It is crucial to keep patient’s past surgical history in mind during interpretation of the images. An Ultrasound, CT and/or MRI of abdomen and pelvis can be used to demonstrate the presence of DGs in the abscess.^3^ There is a dilemma in interpretation of DGs with peritoneal metastasis however history of past LC and presence of calcified material in the abscess favours the diagnosis of DGs.^3,4,6^

These findings were discussed in a multidisciplinary team meeting and the appearance was felt to be due to those of a dropped gallstone and/or appendicolith with resultant abscess formation. Moreover, it was recommended that percutaneous drainage would not solve the problem and a surgical drainage with extraction of the stone would be required as it will prevent the recurrent infection and less dependency on antibiotics leading to rapid recovery.^3,5^

**Figure 4.**
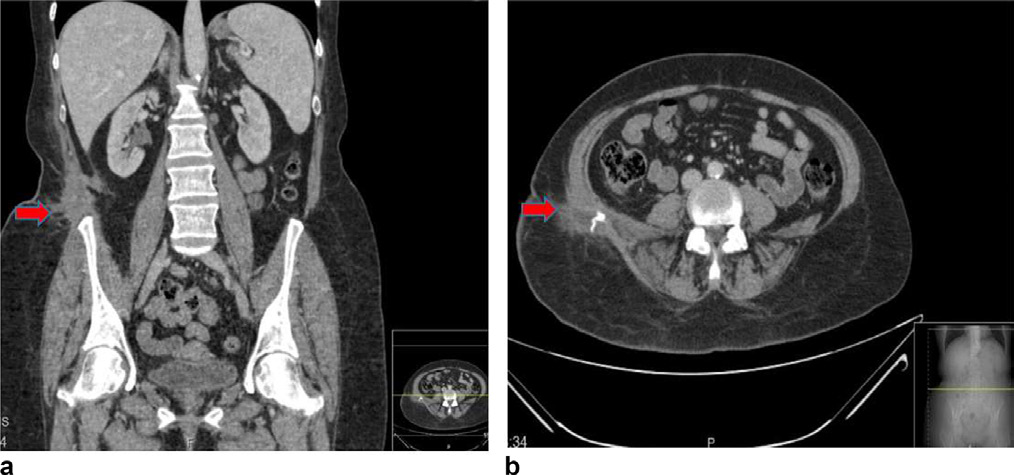
CT abdomen and pelvis with contrast 4 weeks after the procedure showing a small right residual collection in posterolateral abdominal wall/flank with no new collection or free fluid (red arrow).

Subsequently, surgical drainage under general anaesthesia was performed. A large abscess cavity was found subcutaneously with intra-abdominal extension. In addition, a calcified object within the abscess was retrieved from the region of the paracolic gutter. A subsequent histopathology report of the object revealed pigmented, calcified material with neutrophil infiltration consistent with a faecolith and/or a spilled gallstone from previous LC. The pus swab culture showed *Staphylococcus aureus* organism (sensitive to Erythromycin, Penicillin and Flucloxacillin). On follow-up CT, abdomen and pelvis with contrast (4 weeks post-procedure) a small right residual collection was seen posterolateral abdominal wall/flank with no new collection or free fluid (Figure 4a,b).

The DGs/appendicolith can be also be treated with other radiological procedures, *i.e*. catheter drainage and percutaneous CT-guided basket extraction. The cases which fail percutaneous intervention do require surgical intervention.^9^ However, the abscess formation due to DGs/appendicolith is a more common complication (as in our case study) and requires surgical treatment.^5^

## Conclusion

It is important to consider intra-abdominal abscess formation due to a spilled gallstone and/or appendicolith even years after the surgical procedure. The radiologist plays a key role in recognising these complications, and therefore it is important to familiarise with imaging findings.

## Learning points

- Apart from recognising the pathology, another important point in the management is that tube drainage does not work: one has to go in and retrieve the stone, otherwise the abscess will return.

## Final diagnosis

Abscess formation due to dropped gall stones and/or appendicolith.
